# Impact of delayed diagnosis and treatment on tuberculosis infection within families: A case report

**DOI:** 10.1097/MD.0000000000037406

**Published:** 2024-03-15

**Authors:** Jian-Jun Liu, Yin-Ping Feng, Zhong-Da Liu, Jing Guo

**Affiliations:** a Department of Tuberculosis, Lishui Hospital of Traditional Chinese Medicine, Lishui, China

**Keywords:** Case report, delayed diagnosis, delayed treatment, tuberculosis (TB)

## Abstract

**Background:**

A 32-year-old male patient was diagnosed with a 30% left pneumothorax on November 5, 2020, during which chest imaging indicated abnormalities. Despite this, pulmonary tuberculosis (TB) was not diagnosed or treated at that time due to a negative result in the MGIT960 culture. The patient experienced symptoms of cough and expectoration on April 24, 2022. Upon repeating the chest imaging, the condition had worsened, confirming the presence of pulmonary TB, leading to the patient’s hospitalization. On September 1, 2022, the 11-year-old daughter of the patient was diagnosed with pulmonary tuberculosis accompanied by bronchial tuberculosis and tuberculous pleurisy.

**Methods:**

The diagnosis of pulmonary tuberculosis was confirmed through sputum smears and Gene Xpert MTB/RIF testing, for the patient and his 11-year-old daughter in 2022. The patient underwent a 6-month combination therapy (2HRZE/4HR) comprising isoniazid, rifampicin, pyrazinamide, and ethambutol. His daughter with pulmonary tuberculosis accompanied by bronchial tuberculosis and tuberculous pleurisy underwent a 12-month combination therapy.

**Results:**

Late diagnosis and treatment delays contribute to tuberculosis infections within families. Fortunately, after more than 3 months of antituberculosis treatment, the patient experienced relief from cough and sputum secretion, and there was improvement observed in the chest CT scan. Six months later, the patient was successfully cured of TB. 12 months later, his daughter also was successfully cured of TB.

**Conclusion subsections:**

Early diagnosis and treatment of tuberculosis (TB) is vital to reduce transmission, morbidity, and mortality.

## 1. Introduction

The respiratory system is the primary means of transmission for tuberculosis (TB), a chronic infectious disease.^[1]^ It is a significant social and public health problem in the world. According to the World Health Organization (WHO) Global Tuberculosis Report 2022,^[2]^ the estimated number of new TB cases in China in 2021 was 780,000 (down from 842,000 in 2020), with an estimated incidence of 55/100,000 (down from 59/100,000 in 2020). The phenomenon of delayed diagnosis and treatment of patients with TB is widespread.^[3]^ Prolonged delays in diagnosis and treatment lead to patients carrying infectious *Mycobacterium tuberculosis* (MTB) for an extended duration, significantly raising the risk of TB transmission. Patients who experience concurrent delays in diagnosis and treatment may not receive the highest care at the optimal time, may not have good prognosis, and an increased risk of mortality.^[4]^ According to the fifth epidemiological sample survey of TB conducted across China, only 47% of patients with TB in China seek medical treatment in time.^[5]^ Hence, both national and international experts on TB have underscored the significance of studying delays in healthcare seeking among patients with TB. Understanding the delay in the care of patients with TB, analyzing its influencing factors, advising policy makers to take corresponding measures, early detection of patients with active TB infection, and timely treatment measures are conducive to the prevention and control of TB. In this report, we present a case of TB with delayed diagnosis and treatment, emphasizing the importance of increased awareness among doctors treating TB infections.

## 2. Case reports

### 2.1. Chief complaints

A 32-year-old male patient was diagnosed with a 30% left pneumothorax on November 5, 2020, during which chest imaging indicated abnormalities. Despite this, pulmonary TB was not diagnosed or treated at that time due to a negative result in the MGIT960 culture. The patient experienced symptoms of cough and expectoration on April 24, 2022. Upon repeating the chest imaging, the condition had worsened, confirming the presence of pulmonary TB, leading to the patient’s hospitalization.

This study was conducted with approval from the Ethics Committee of Lishui Hospital of Traditional Chinese Medicine(2023-LW-058). Written informed consent was obtained from the minor legal guardian for the publication of any potentially identifiable images or data included in this article.

### 2.2. History of present illness

On April 24, 2022, the patient sought medical attention due to a persistent cough and expectoration lasting for 3 months. His chest imaging revealed irregularly shaped, nodular, and strip-like shadows with blurred edges and uneven densities in the right upper lobe apex segment, the left upper lobe apex posterior segment, tongue segment, and the dorsal segment of the lower lobe. Multiple thin-walled cavities were seen in the left upper lobe of the lung, and the inner wall was smooth. The sputum was purulent and yellow in color. The diagnosis of pulmonary TB was confirmed through 3 consecutive sputum smear tests, showing acid-fast bacilli with intensities of 2+, 2+, and 3+. Additionally, the Sputum Gene Xpert MTB/RIF results were positive for TB DNA and negative for the rifampicin resistance gene rpoB. Furthermore, the MGIT960 culture was positive for mycobacterium, affirming the diagnosis. The patient was subsequently treated with 2HRZE/4HR.

### 2.3. History of past illness

The patient was admitted to our hospital on November 5th, 2020. The patient’s left lung pneumothorax was promptly addressed on the second day with antimicrobial medication and oxygen inhalation therapy. On November 9, 2020, chest CT revealed scattered nodular, flocculent, and cable-like shadows with increased density in the upper lobe of the left lung, with poorly defined boundaries, and calcification in the lesion. On November 10, 2020, a comprehensive bronchoscopy was conducted, revealing no abnormalities upon microscopic examination. The MGIT960 culture of the left upper lobe lavage fluid tested negative for Mycobacterium; however, the Tuberculin Skin Test (TST) yielded a positive result. It is important to note that, at that time in 2020, the patient had not been diagnosed with TB and had not initiated anti-TB therapy.

### 2.4. Personal and family history

There were no abnormalities noted in the patient’s personal or family history.

### 2.5. Physical examination

After a physical examination, the following parameters were recorded as follows: temperature was 36.5 °C, pulse rate was 85 bpm, blood pressure was 108/69 mm Hg, and breathing rate was 20 breaths per minute. Chest examination revealed normal chest, symmetrical bilateral respiratory activity, symmetric fibrillation, resonant percussion sounds in both lungs, coarse breath sounds in both lung fields, and no discernible dry or wet rales.

### 2.6. Laboratory investigation

Sputum smear tests were conducted 3 times, revealing the presence of acid-fast bacilli with intensities of 2+, 2+, and 3+. Additionally, the Sputum Gene Xpert MTB/RIF results were positive for TB DNA and negative for the rifampicin resistance gene rpoB. Furthermore, the MGIT960 culture tested positive for Mycobacterium.

### 2.7. Imaging examination

**Figure 1.**
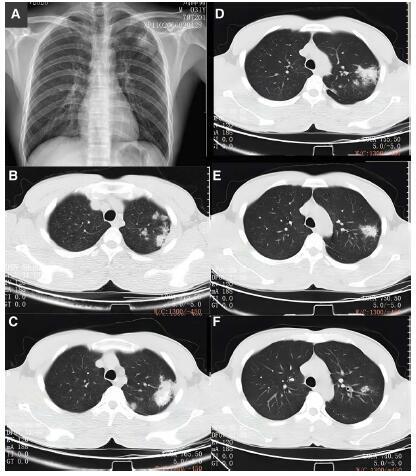
Chest X-ray of the patient on November 6, 2020 and Chest CT of the patient taken on November 9, 2020.

**Figure 2.**
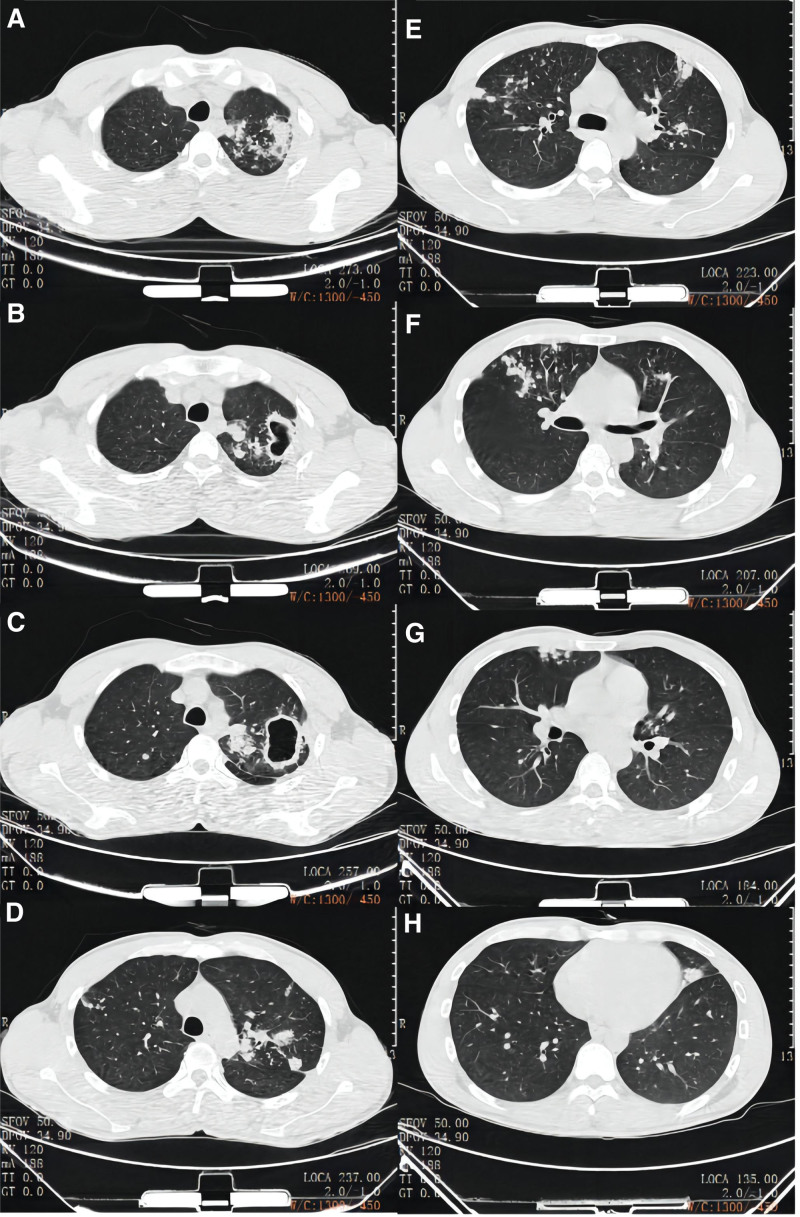
Chest CT of the patient taken on May 5, 2022.

On November 9, 2020, his chest CT revealed scattered nodular, flocculent, and cable-like shadows with increased density in the upper lobe of the left lung, with poorly defined boundaries, and calcification in the lesion (Fig. 1). His chest CT scan, performed about 18 months later, revealed irregular density, nodular and strip-like shadows with blurred edges and dispersed thickening in the tongue segment, the dorsal segment of the lower lobe, the right upper lobe apex segment, and the left upper lobe apex posterior segment. On May 5, 2022, there were several thin-walled cavities visible in the left upper lobe of the lung, and the inner wall was smooth (Fig. 2).

### 2.8. Final diagnosis

The patient was diagnosed with TB.

### 2.9. Treatment

The patient underwent a six-month combination therapy (2HRZE/4HR) comprising isoniazid(H), rifampicin(R), pyrazinamide(Z), and ethambutol(E).

### 2.10. Outcome and follow-up

**Figure 3.**
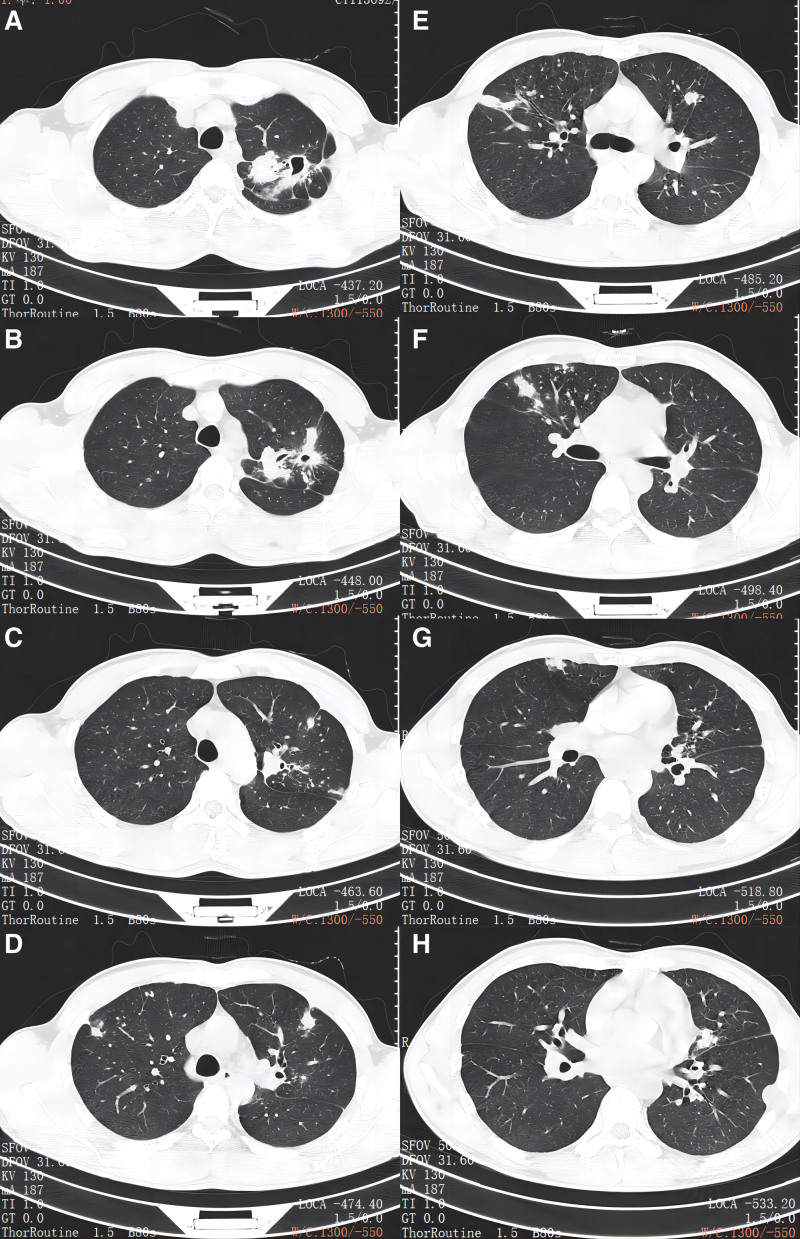
Chest CT of the patient taken on August 29, 2022.

**Figure 4.**
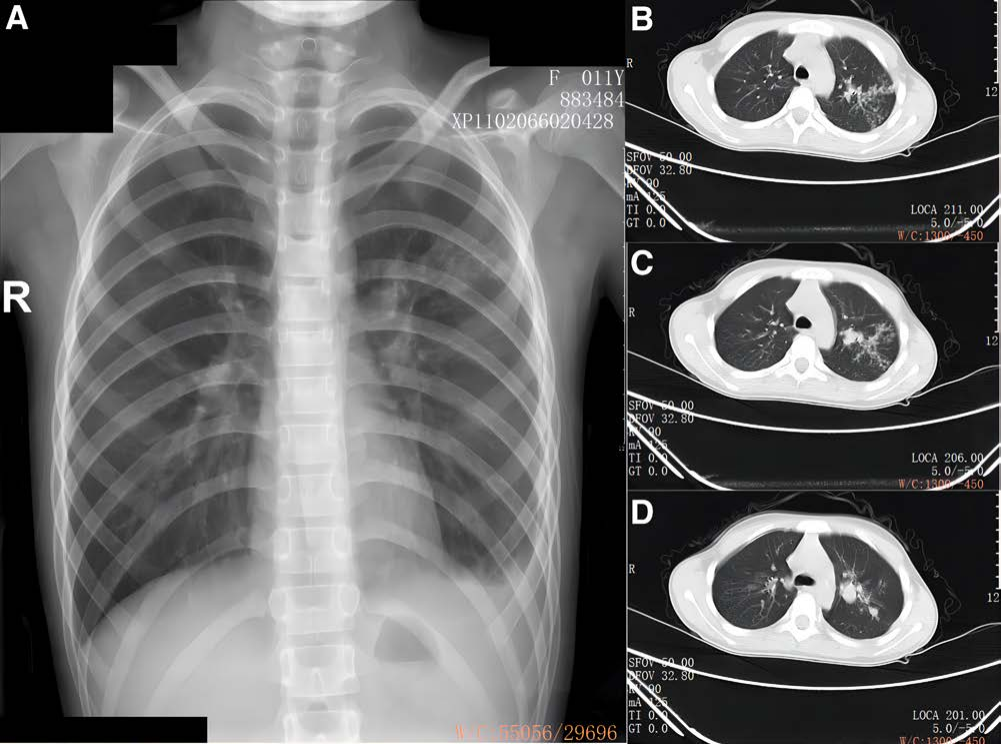
Chest X-ray and chest CT of the patient’s daughter taken on August 30, 2022.

Fortunately, after more than 3 months of antituberculosis treatment, the patient experienced relief from cough and sputum secretion, and there was improvement observed in the chest CT scan (Fig. 3). Six months later, the patient was successfully cured of TB. Unfortunately, on September 1st, 2022, the patient’s 11-year-old daughter was diagnosed with pulmonary TB accompanied by bronchial TB and tuberculous pleurisy. Patchy and nodular high-density shadows and pleural effusion were visible in her left lung (Fig. 4). Bronchoscopy showed swelling and White necrosis at the opening of the appropriate segment of the left upper lobe of the lung. There was no adverse and unanticipated events.

## 3. Discussion

Diagnosis delay, also known as “total diagnosis delay,” is the amount of time that passes between the first symptoms of the patient and their TB diagnosis. This time span includes 2 stages: delay in seeing a doctor and delay in diagnosis. Among them, the “delay in seeing a doctor” refers to the time interval between the first symptom of a patient and the first visit to a medical institution. The “delay in diagnosis” refers to the time required from the first visit to the medical institution until the patient is diagnosed with TB.^[6]^ As for the interval of delay, it is generally believed that the delay of visit and diagnosis exceeds 2 weeks (14 days) respectively. A study conducted in India revealed that the two-week window period between the onset of symptoms and the initial screening was more sensitive, a finding which was acknowledged by the WHO.^[7]^ China has 1 of the highest rates of tuberculosis infections among middle and high-income nations, and it is also 1 of the few such countries. In China, the “delayed doctor visits” refers to the time interval between the onset of symptoms and the initial visit of a patient that exceeds 2 weeks.^[8]^ Although TB prevention and control in China has achieved some results, according to some studies, more than 50% of patients may still not see a doctor in time.^[5]^ A study conducted in Nanjing revealed that the average delay in diagnosis of patients with TB was 50.3 days.^[9]^

TB infection results from delayed diagnosis and treatment. Delays in diagnosis and treatment worsen the prognosis of TB, and sustain TB transmission in the community, making TB elimination a formidable task, especially in these countries. In 2018, nearly one-third of the population with TB was estimated to be undiagnosed globally.^[10]^ The delay in diagnosis and treatment is detrimental to the prognosis and perpetuates TB transmission in the community, and thus poses a great challenge to eliminate TB.^[11]^ Clinically, the majority of patients with TB frequently undergo a protracted period ranging from weeks to months between the onset of symptoms and diagnosis. Delays in diagnosis can have 2 consequences: on 1 hand, they can cause the disease to worsen and miss the best opportunity for early diagnosis and treatment; on the other hand, they can spread TB across the community. Timely detection of TB is a key requirement for improving survival of patients.

In several nations and locations, delayed diagnosis and treatment of TB have been linked to socio-demographic, clinical health system, and economic aspects, as supported by a collective body of recent systematic reviews.^[12]^ Moreover, the prolonged bacterial replication in patients often leads individuals with positive sputum tests to transition from moderate illness to severe conditions in clinical practice. Such cases are further complicated by delayed diagnoses. Enhancing diagnostic capabilities for patients with negative sputum smears is essential. More attention should be paid to the delayed diagnosis and treatment of patients with positive etiology. Multiple referrals for patients not only result in delayed diagnosis but can also lead to delays in treatment and increase disease transmission.

To develop treatment plans to significantly lower the prevalence of TB, it is essential to determine the factors that contribute to the delayed TB diagnosis and treatment. Ensuring early TB diagnosis is a part of the WHOs End TB plan, which aims to reduce TB mortality by 75% and TB incidence in the general population by 50% by 2025.^[13]^ Enhancing the diagnosis and treatment capabilities of healthcare facilities and medical personnel is vital, but so is increasing the public awareness of the fundamentals of TB. At the same time, it is recommended that primary medical institutions incorporate TB diagnosis techniques such as chest X-ray, sputum smear for acid-fast bacilli, and PPD test in the annual physical examination of the elderly in rural areas, so as to improve the accessibility of TB prevention and treatment services, identify patients in a timely manner, and carry out early intervention to shorten the delay in diagnosis and treatment of patients with TB, and increase the treatment success rate. Numerous studies on delayed doctor visits have been carried out in recent years, and it has been found that there are a variety of reasons why the visits are delayed. However, relatively little research has been conducted on intervention measures for delayed diagnosis. On the basis of an understanding of the specific reasons for the delay in different populations in different regions, it is recommended that in the future, targeted intervention should be carried out to reduce the incidence of delayed diagnosis, strengthen the multi-sectoral cooperation mechanism, effectively prevent, control, and manage TB, and provide the foundation for the promotion of TB prevention and treatment in China.

In a nutshell, the phenomenon of delayed diagnosis is widespread in all countries, especially in patients with immunosuppressive diseases and who are resistant to drugs. They include not only personal factors of the patients, but also the delay in the diagnosis caused by insufficient knowledge and technological infrastructure of medical institutions. To address these issues, it is imperative to first increase awareness of TB while also enhancing the ability of medical facilities and medical staff to diagnose and treat patients. With the extensive research on the delay in diagnosis in various countries, it was discovered that the factors leading to the delay are diverse and different from country to country and region to region, which provide a strong basis for improving the problem of diagnosis delay based on the actual situation. However, there is less research on intervention strategies for delayed diagnosis at this stage. It is recommended to carry out targeted intervention based on understanding the specific causes of delay in different regions and populations in the future, in order to reduce the incidence of delayed diagnosis and strengthen the prevention and control of TB.

## 4. Limitations

The limitation of this case is that there is no molecular confirmation that M. tuberculosis infected in this patient is homologous to M. tuberculosis infected with his daughter. And this study did not analyze the reasons leading to the delay in diagnosis in this patient.

## 5. Conclusion

To prevent transmission, morbidity, and mortality from TB infection, early diagnosis and treatment are crucial. This case study confirmed that delayed diagnosis and treatment can lead to TB infections. Furthermore, it advances medical comprehension and alert mechanisms within the field of TB treatment in China. Additional prospective studies investigating the effects of delayed diagnosis and treatment are necessary.

## Acknowledgements

This study was supported by a grant from Lishui tuberculosis clinical medical research center. And none of the authors have any financial disclosure or conflict of interest.

## Authors contributions

**Conceptualization:** Jian-Jun Liu,Jing Guo.

**Data curation:** Yin-Ping Feng,Jian-Jun Liu.

**Formal analysis:** Zhong-Da Liu,Yin-Ping Feng, Jing Guo.

**Funding acquisition:** Zhong-Da Liu

**Roles/Writing – original draft:** Jian-Jun Liu,Jing Guo.

**Writing – review & editing:** Zhong-Da Liu,Jing Guo.
